# Cell-in-cell: a potential biomarker of prognosis and a novel mechanism of drug resistance in cancer

**DOI:** 10.3389/fonc.2023.1242725

**Published:** 2023-08-10

**Authors:** Xiaona Liu, Jun Yang

**Affiliations:** Department of Pathology, The Second Affiliated Hospital, Xi’an Jiao Tong University, Xi’an, Shaanxi, China

**Keywords:** cell-in-cell, homotypic cell-in-cell, heterotypic cell-in-cell, prognosis, diagnosis, anticancer treatment, drug resistance

## Abstract

The cell-in-cell (CIC) phenomenon has received increasing attention over recent years because of its wide existence in multiple cancer tissues. The mechanism of CIC formation is considerably complex as it involves interactions between two cells. Although the molecular mechanisms of CIC formation have been extensively investigated, the process of CIC formation remains ambiguous. Currently, CIC is classified into four subtypes based on different cell types and inducing factors, and the underlying mechanisms for each subtype are distinct. Here, we investigated the subtypes of CIC and their major mechanisms involved in cancer development. To determine the clinical significance of CIC, we reviewed several clinical studies on CIC and found that CIC could serve as a diagnostic and prognostic biomarker. The implications of CIC on the clinical management of cancers also remain largely unknown. To clarify this aspect, in the present review, we highlight the findings of recent investigations on the causal link between CIC and cancer treatment. We also indicate the existing issues that need to be resolved urgently to provide a potential direction for future research on CIC.

## 1 Introduction

**Figure 1:**
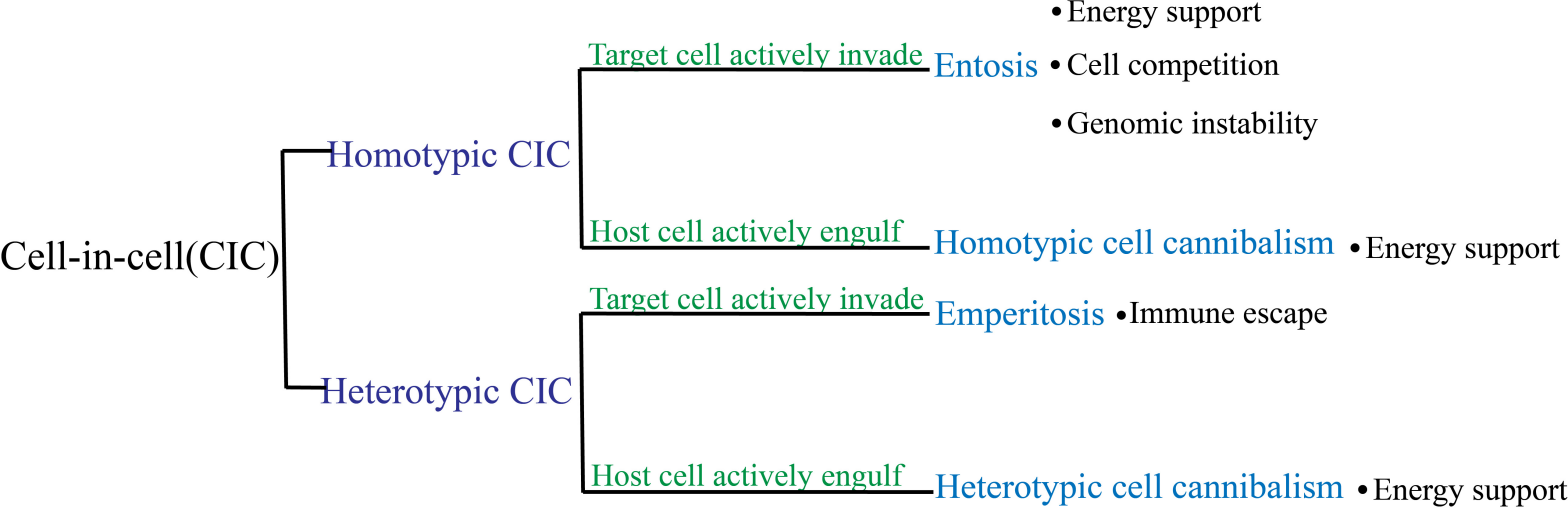
Classification of cell-in-cell (CIC) subtypes and their roles in tumors. Based on the type of internalized cell, CIC is categorized as homotypic or heterotypic. These categories are further divided into two subgroups depending on whether the target cells or the host cells initiated the process: entosis and homotypic cannibalism for homotypic CIC, and emperitosis and heterotypic cannibalism for heterotypic CIC.

Cell-in-cell (CIC) is a structure form wherein one viable cell is internalized into another cell, i.e., the internal cell (referred to as the target cell) is enclosed within a large vesicle in the external cell (referred to as the host cell) (1–3). The presence of lymphocytes within intestinal epithelial cells was first reported in 1864 (4), which led to the discovery of cell-in-cell structures (CICs). Despite being identified over a century ago, CIC has been traditionally viewed as a pathological feature that is primarily investigated by researchers in the field of pathology. With the advancements in microscopy techniques and cellular microscopic examination methods, an increasing number of CICs have been observed in both pathological and physiological conditions, with their most frequent occurrence being in cancer tissues (5). Thus far, numerous studies have explored the significance and mechanisms of CIC in diverse biological processes. CIC plays a crucial role in embryonic development, viral infection, immune homeostasis in the liver, and tumor development. Given the escalating rate of cancer-related mortality in recent years, coupled with the intricate underlying mechanisms of tumorigenesis, our focus is on the classification and significance of CICs in cancer. Although the formation of cell–cell interactions results in CIC structures with similar appearances, the classification of CICs into homotypic and heterotypic CICs is determined by the target cell types involved (**Figure 1**). Homotypic CICs are formed through internalization among tumor cells, where one tumor cell is internalized by another, whereas heterotypic CICs are formed by the internalization of immune cells into tumor cells whereby an immune cell resides within a tumor cell. There are distinct molecular mechanisms involved in the initiation of target-cell internalization into host cells among different subtypes of CIC. Most target cells within CICs are degraded to facilitate tumor progression through, for example, energy reprogramming (6) and immune escape (7). Therefore, it can be inferred that CICs are closely related to tumor diagnosis and prognosis.

Chemoradiotherapy, immunotherapy, and molecular-targeted drugs have emerged as promising therapeutic modalities for preventing tumor recurrence. However, despite these advancements in tumor treatment, a significant number of patients still exhibit non-response and drug resistance to these therapies (5, 8, 9). The mechanisms of non-response to tumor therapy and drug resistance, including the effects of CIC on anticancer agents, have been extensively studied. To date, however, no review has been conducted on the implications of CIC in cancer treatment. In the present article, we review the impact of CICs on anticancer therapy and aim to provide evidence for future research and the clinical treatment of CIC in tumors.

## 2 CIC structure: past and present understanding

CICs were first observed in tumors by Steinhaus in 1891 (10). In the following year, the phenomenon of tumor cells engulfing other tumor cells was also reported by H. Stroebe (11). Because this phenomenon was identical to phagocytosis, it was referred to as “cell phagocytosis” (12). Subsequently, Leyden described the CIC phenomenon in the tumor as a “bird’s eye cell” and termed it “cell cannibalism,” wherein a tumor cell with a large crescent-shaped nucleus contains a small tumor cell in its cytoplasm (13). In 1925, Lewis first experimentally demonstrated the viability of internalized cells in CIC; this enabled the differentiation of CIC from phagocytosis, in which the phagocytes internalize only dead cells or dying cells (3). Later, Ugini et al. found that metastatic melanoma cells could engulf lymphocytes. This phenomenon was described as “heterotypic cell cannibalism,” whereas the cell cannibalism process occurring between two tumor cells was termed “homotypic cell cannibalism” (14). In 1956, Humbel observed that lymphocytes could invade tumor cells and yet remain intact; the author termed this phenomenon “emperipolesis”, which is a Greek word meaning “inside round about wandering” (15). Presently, emperipolesis is often used as a general term to describe the movement of a living cell into another cell; this is because a variety of specific names have been used according to the different mechanisms involved in CIC formation (5). In 2007, a non-apoptotic mechanism of programmed cell death was discovered; this process was termed “entosis,” a Greek word meaning “inside” or “into”. In this process, once the cells detach from the matrix, they actively invade the neighboring host cells, thereby inducing the host cells to undergo degradation or release from the matrix (16). In 2013, Wang et al. demonstrated that live cytotoxic immune cells were engulfed by tumor cells, and the phenomenon of internalized cell death occurred through apoptosis; the authors termed this phenomenon “emperitosis,” which was derived from “emperipolesis” and “apoptosis” (17).

## 3 Mechanisms and consequences

### 3.1 Heterotypic CIC

Heterotypic CIC in tumors refers to the engulfment of immune cells by tumor cells and involves heterotypic cell cannibalism and emperitosis.

**Figure 2:**
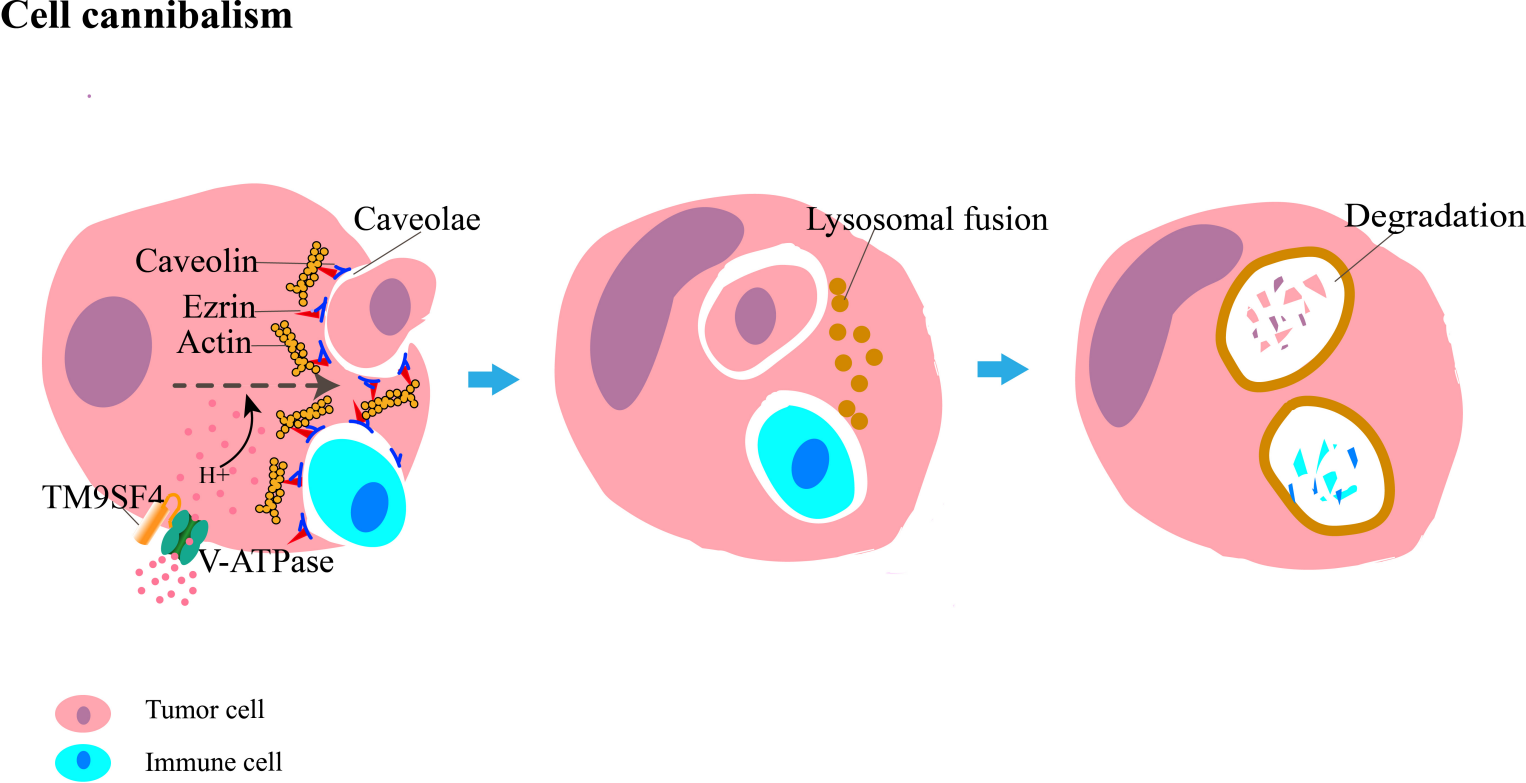
Diagrammatic representation of cannibalism, in which tumor cells engulf their sibling or immune cells. The main molecules involved are caveolin, ezrin, and TM9SF4. Caveolin interacts with actin through ezrin to invaginate the caveolae, which promotes the internalization of the target cell. The co-localization of TM9SF4 and V-ATPase allows H^+^ to enter the cell. The acidic environment within the cell enhances cannibalistic activity. After engulfment is completed, target cells are eventually degraded by lysosome-mediated cell death.

The main molecular components of cell cannibalism include caveolins, ezrin, and transmembrane 9 protein (TM9) (**Figure 2**). Caveolins, the main protein components of caveolae, are a family of integral membrane proteins composed of three members: caveolin-1 (CAV-1), CAV-2, and CAV-3. Caveolin-1 mediates selective endocytosis by forming microcapsule-like structures during early cell contact; this process is blocked by anti-caveolae antibodies that inhibit the development of cell cannibalism (18). Ezrin is a member of the ezrin–radixin–moesin family, and it mainly acts as a linker between the cytoskeleton and the cell membrane in epithelial cells, maintains cell morphology and movement, binds adhesion molecules, and regulates signal transduction (19). Further studies have shown that ezrin functions as a modifiable link between caveolin-1 and actin (14, 20), wherein ezrin and caveolin-1 interact with each other and activate actin, which is associated with cell motility and plays a key role in the formation of cannibalistic vacuoles and progression of cell cannibalism. TM9 belongs to a highly conserved family of transmembrane proteins; it was identified as a protein that regulates efficient cell-to-cell adhesion and phagocytic process (21). In humans, the TM9 superfamily comprises four proteins (TM9SF1–4). TM9SF4 is highly expressed in cannibalistic cells, whereas its knockdown inhibits cell cannibalism (22). The colocalization of TM9SF4 and V-ATPase increased intracellular and extracellular pH, whereas TM9SF4 knockdown decreased intracellular pH and increased extracellular pH (23). Thus, TM9SF4 might play a role in maintaining an acidic tumor microenvironment in which metastatic tumor cells can survive and contribute to tumor metastasis. TM9SF4 can also activate some lytic enzymes involved in cannibalistic activity, such as cathepsins and other proteases, in an acidic environment, which promotes the development of cell cannibalism.

**Figure 3:**
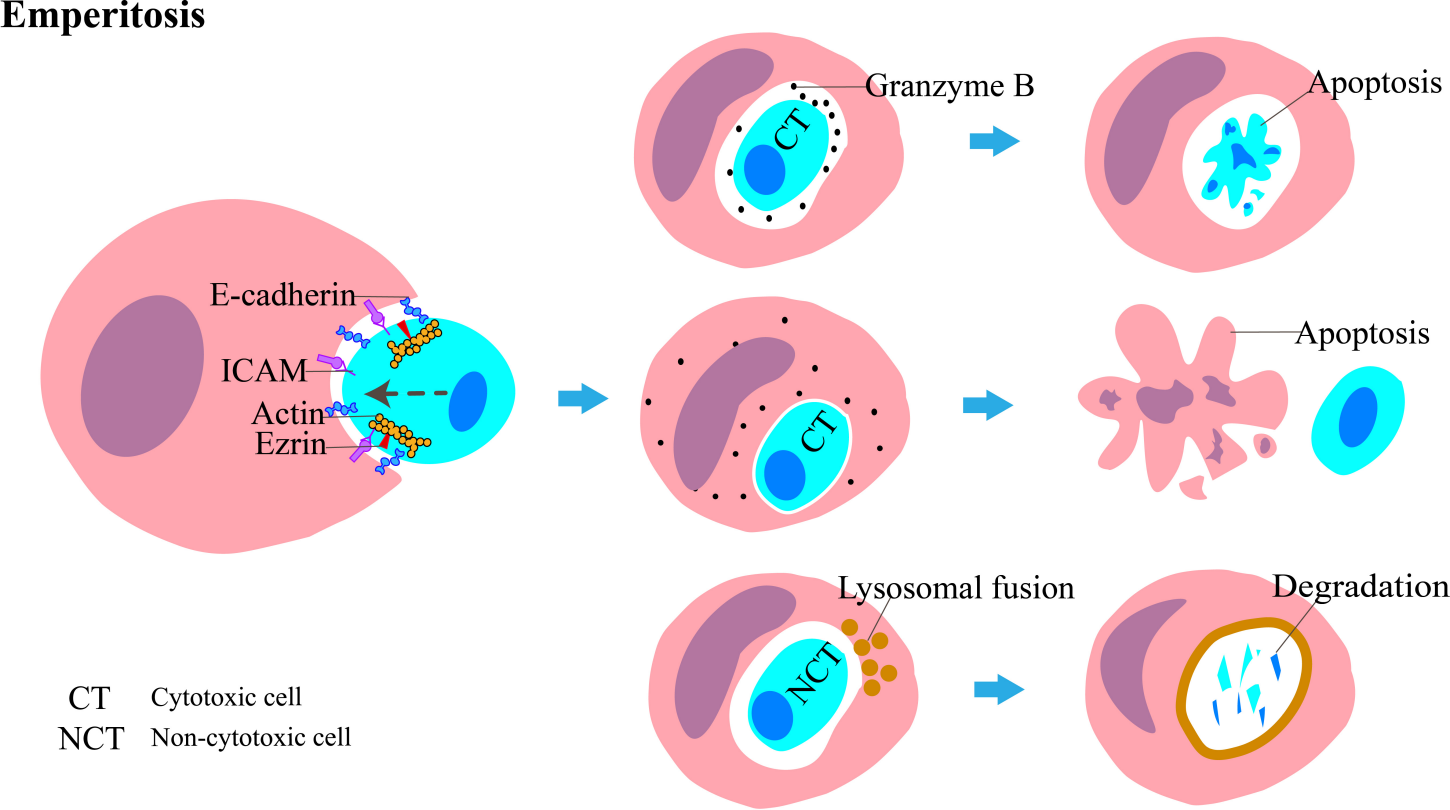
Diagrammatic representation of emperitosis, in which immune cells invade into tumor cells. The main molecules involved in this process are ICAM, ezrin, and E-cadherin. The invading immune cells are categorized into cytotoxic cells and non-cytotoxic cells. The vesicles encapsulating the internalized cytotoxic cell expand rapidly. Granzyme B is absorbed by cytotoxic cells and leads to apoptosis. Reciprocally, granzyme B is released into the host cell cytoplasm and induces apoptosis. The non-cytotoxic cell dies following lysosome-mediated degradation.

To the best of our knowledge, the primary molecules involved in eperitosis include ezrin, E-cadherin, and ICAM (**Figure 3**) (24). IL-6 increases cell adhesion by upregulating ICAM to activate the STAT3/5, ERK, and Rho-ROCK signaling pathways; enhances cell motility; and promotes the development of emperitosis (7).

The engulfed immune cells usually die. Cytotoxic immune cells such as CD8^+^ T cells, natural killer (NK) cells, cytokine-induced killer (CIK) cells, and lymphokine-activated killer (LAK) cells are internalized by tumor cells; the released granzyme B cannot enter the cytoplasm of the tumor cell through vesicles and is taken up again by cytotoxic cells, which leads to their apoptosis, whereas non-cytotoxic cells, such as B cells, undergo degradation through the lysosomal-dependent pathway (17). Reciprocally, some NK cells enter tumor cells and kill them while residing inside them, and this mode of killing is even more efficient than their “kiss-killing” method, carried out from outside of the tumor cells (25). The conditions under which these two distinct outcomes of NK cells occur, however, remain ambiguous.

### 3.2 Homotypic CIC

Homotypic CIC comprises homotypic cell cannibalism and entosis; the mechanism of the former process has been discussed above.

**Figure 4:**
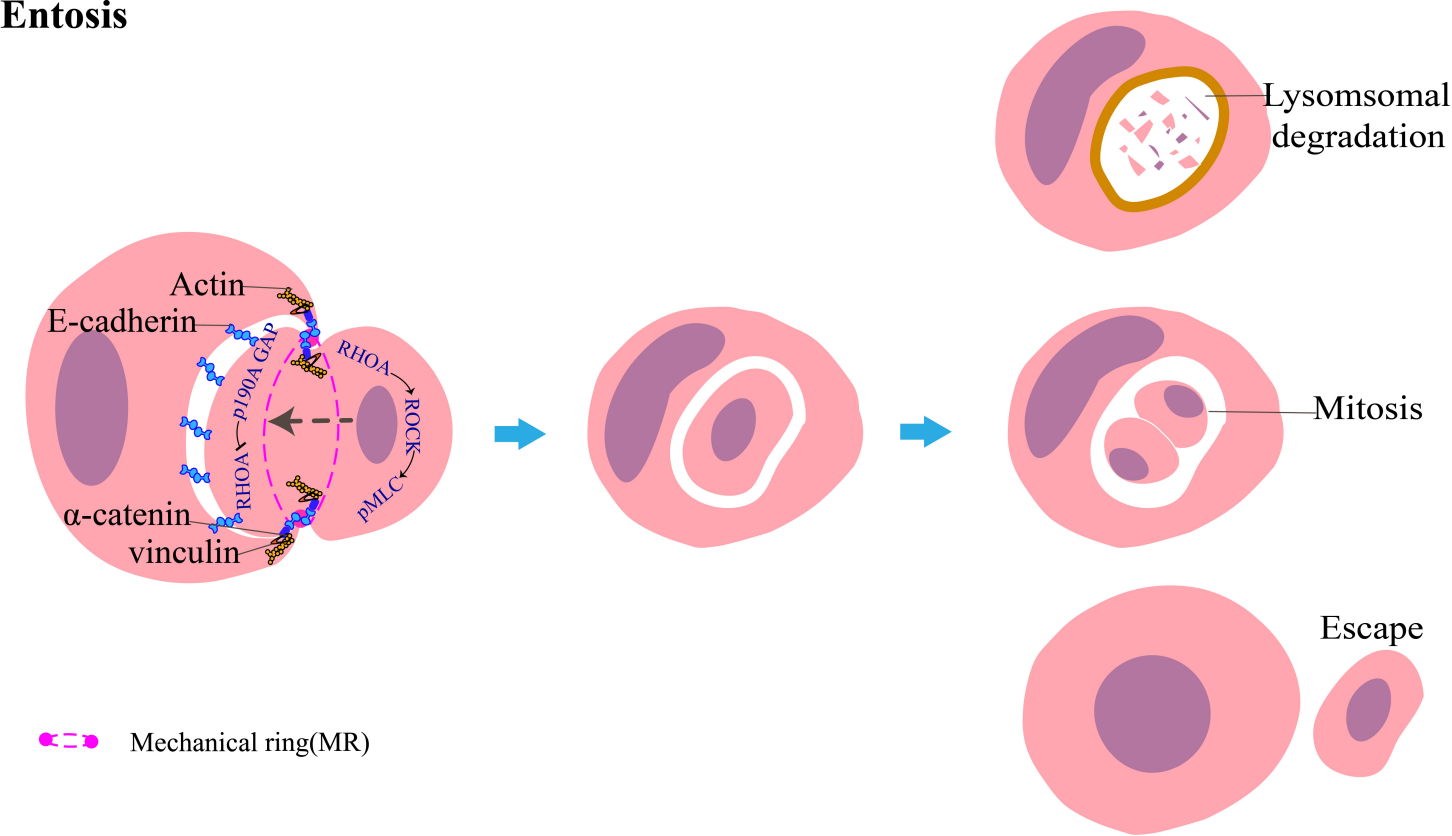
Diagrammatic representation of entosis: target tumor cells invade host tumor cells. The host and target cells adhere to each other through E-cadherin, which recruits p190A GAP. This inhibits RhoA at this interface. Conversely, the activation of the RHOA-ROCK-pMLC pathway promotes actomyosin contraction at the rear of the invading tumor cell, leading to the generation of forward momentum. Cell adhesion and actomyosin contraction coordinate through a cyclic mechanical complex mainly composed of vinculin, α-catenin, E-cadherin, and actin. The target cells are mainly degraded by the lysosome-mediated cell death pathway; a minor portion of the target cells can survive and undergo mitosis within the host cell or even escape from the host cell and remain intact.

Entosis is mainly associated with cytoskeletal changes. The most important factor in entosis is the Rho family (16), which belongs to the small GTPase family and includes Rho (RhoA, RhoB, and RhoC), Rac (Rac1, Rac2, and Rac3), and Cdc42. These three proteins act as switches in the GTP-bound conformation and coordinate cell adhesion and metastasis. RhoA activates the downstream molecule ROCK in target cells, which phosphorylates myosin light chain (MLC) and inhibits MLC phosphatase, thereby promoting the contraction of actomyosin (26). Following the recruitment of E-cadherin and/or P-cadherin complexed with intracellular catenin for adherence to the host cell and the target cell, Rho-GAP enzymes, such as p190A GAP, are recruited at the contact interface between the two cells, thereby inactivating GDP conformation and inhibiting RhoA activity. This allows the formation of a gradient across the cell (27, 28), which results in the accumulation of RhoA in the tail of the internalizing cell contrary to the cell adhesion site, thus generating a forward driving force to promote the invasion of the target cell (16). Cell adhesion and actomyosin contraction are spatially independent processes that are coordinated by a complex multimolecular mechanical ring (MR) (29). Following the contraction of cortical actomyosin linked to the adhesion junction, this mechanical complex recruits the activated vinculin, which senses the mechanical force across the paired cells engaged in entosis (**Figure 4**). Vinculin is a force-sensitive protein comprising three regions: an acidic N-terminal domain (head), a proline-rich middle segment (neck), and a basic C-terminal domain (tail). Vinculin exists in the cell in two conformations: an open, active form and a closed, auto-inhibited state in which the head domain forms extensive interactions with the tail (30, 31). The open form of vinculin can stabilize E-cadherin-mediated cell adhesion by enhancing the interaction between α-catenin and F-actin. During CIC formation, E-cadherin-mediated cell adhesion is linked to F-actin in the cell cortex through α-catenin/β-catenin. Because F-actin is a component of actomyosin and is linked to α-catenin, the vinculin-binding domain (VBR) of α-catenin is exposed, which enables the head of vinculin to attach the VBR, as the tail interacts with F-actin. Vinculin is thus recruited at MR, which strengthens the link between α-catenin and actin. Finally, it promotes the formation of CIC. The development of entosis is affected by many factors and stimulatory signals, such as AMPK activation in response to glucose starvation (32). UV irradiation promotes the formation of entosis by activating the JNK and p38 signaling pathways (33). De-adhesion between the cells and the matrix downregulates the FAK signaling pathway and induces TRAIL to promote the development of entosis (34). Sterols inhibit entosis development by inhibiting the phosphorylation of MLC (35). IL-8 promotes entosis by upregulating P-cadherin and γ-catenin to increase intercellular adhesion (36).

Internalized cells undergo one of the following three fates: (1) most cells die by degradation through the lysosomal-dependent pathway; (2) approximately 12%–18% of cells escape from the external cells, and the released cells function normally and undergo mitosis; and (3) approximately 0.8%–9% of the inner cells can divide in the cytoplasm of the outer cells (16). However, only disparate outcomes have been observed, and the mechanisms that determine the three distinct internalized cell fates remain unknown.

## 4 The role and function of CIC in cancer

CIC exists in various cancers, thus indicating its relevance in cancer development. Under nutrient-replete conditions, such as glucose starvation and amino acid starvation, the rapid proliferation and metastasis of tumor cells require more energy support; consequently, the tumor cells degrade and digest their sibling or heterotypic cells by engulfing these cells and reusing their metabolites as their own nutrients to promote their survival and proliferation (14, 32, 37). This feature may be related to the reprogramming of tumor metabolism—a hallmark of cancer. Meanwhile, CIC enhances glucose metabolism through the transfer of mitochondria (6), which may contribute to the reprogramming of tumor metabolism—which is also a hallmark of cancer—and promote tumor malignancy. In heterotypic CIC, immune cells are engulfed and eliminated by tumor cells, thereby resulting in the immune escape and survival of tumor cells (38). During CIC formation, the target cells enter the host cells through myosin contraction and are killed. The stiffness of a cell is determined by the structure of its cytoskeleton, with cells that are softer exhibiting greater deformability, a property that determines whether they can traverse through the interstitial space between the fibrous stroma and the narrow endothelium of blood vessels. Therefore, RhoA and actomyosin can determine the deformability of the cell. The host cells have a stronger deformability, whereas the target cells are more rigid. This promotes competition between tumor cells, and the survival of highly deformable cells is associated with a higher metastatic capacity (39). This finding indicates that tumor cells with stronger deformability are more likely to survive and that CIC can facilitate clonal selection during tumor development. The internalized cells can persist in the outer cells and even undergo mitosis; this persistence can interrupt the mitosis of the outer cells, leading to the destruction of the contraction ring and the production of binuclear tumor cells (16). Ultimately, these cell behaviors result in genomic instability and the accumulation of mutations in tumor cells. Genomic instability is a hallmark of tumor development and promotes tumor progression and invasion. Entosis can also eliminate tumor cells detached from the stroma and inhibit their metastasis, which affects tumor suppression. A recent study suggested that genetic transfer, such as cell fusion and phagocytosis of apoptotic bodies, render the acquisition of phenotypes such as tumor aggressiveness and drug resistance. CIC formation between tumor cells results in cell clones associated with the genetic transfer and gain of malignancy by allowing the assimilation of aggressive phenotypes from distinct coexisting subpopulations; this indicates a new mechanism that allows rapid tumor development (40). As mentioned earlier, the promotive or suppressive effects of CIC may be related to the diagnosis and prognosis of tumors.

### 4.1 Diagnostic value

**Figure 5:**
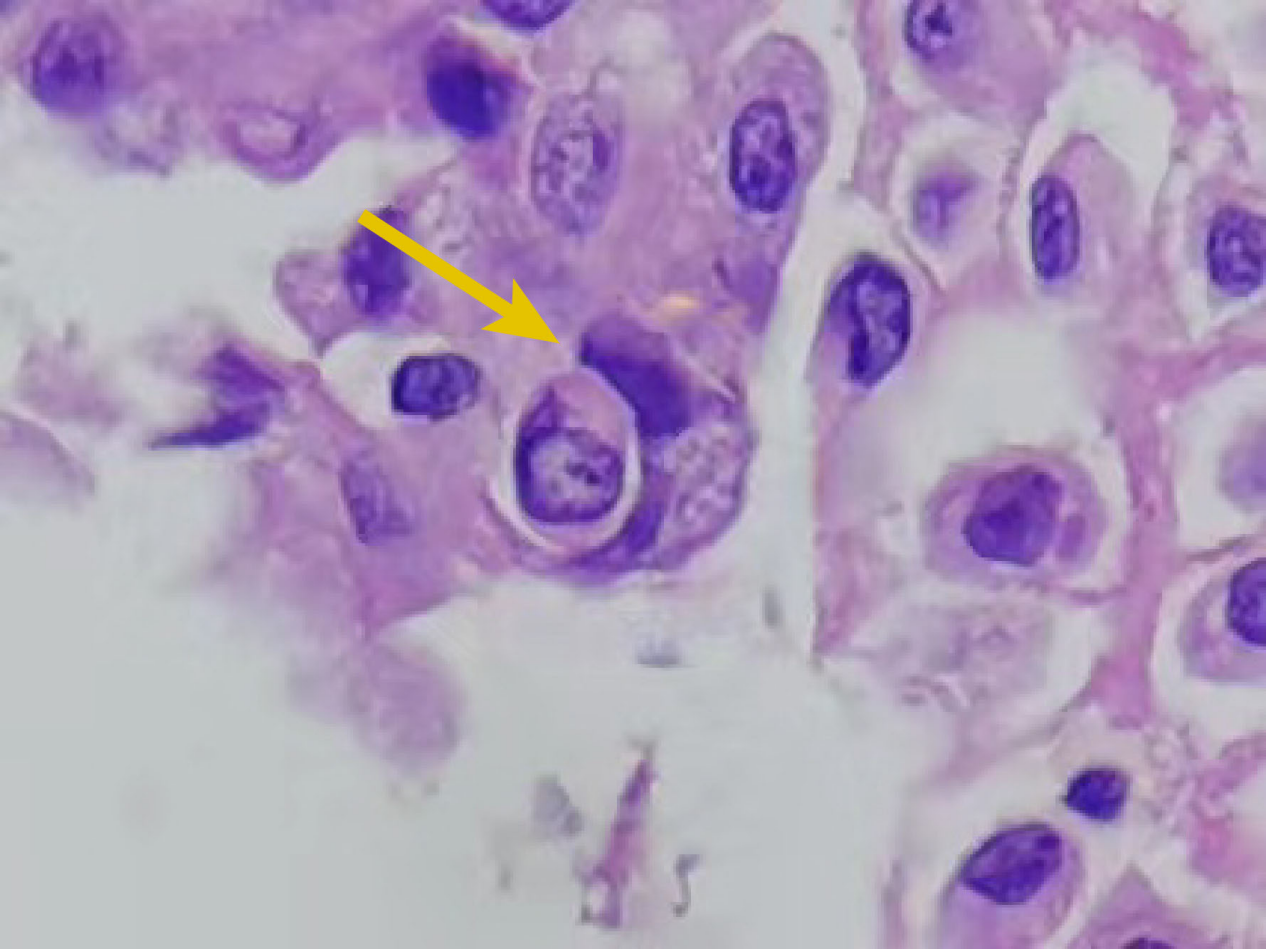
A cell-in-cell (CIC) structure (arrow) in pleural effusion cytology (hematoxylin and eosin staining, × 400). The internal cell is fully enclosed within a vesicle located in the external cell, which features a crescent-shaped nucleus.

A cytological examination is critical for the differentiation of benign and malignant diseases and provides a reliable basis for diagnosing tumors. Malignant cells in effusion fluids show various characteristics such as ball-like three-dimensional clusters, pleomorphism, nuclear hypochromasia, nuclear membrane irregularities, and prominent nucleoli (41). The characteristics are, however, sometimes difficult to differentiate from reactive hyperplasia; hence, more reliable diagnostic features are needed to differentiate between benign and malignant cells in effusion fluids. Kojima et al. suggested that cell cannibalism detected in the urine cytological examination could be an indicator of bladder cancer (42). A further study involving 10 malignant effusion samples, 10 benign effusion samples, 10 malignant urine samples, and 10 benign urine samples showed that the level of cell cannibalism was significantly higher in malignant samples than in benign samples (41), and this finding was consistent with the results of our previous study (43). We collected 98 malignant cell samples (including pleural effusion, ascites, and pericardial effusion) and 30 benign cell samples. The results showed that the detection rate of CIC was 26.5% (26 out of 98). Among these 26 samples, the proportion of CIC cells was 1%–10% in 73.1% (19 out of 26) of samples, 11%–20% in 19.2% (5 out of 26) of samples, and more than 20% in 7.7% (2 out of 26) of samples. The CIC phenomenon was not detected in 30 non-malignant tumor samples. Furthermore, no significant difference was observed in the proportion of CIC cells between the different types of malignant tumors; however, a significant difference was noted between malignant tumors and non-malignant tumors. This finding indicates that although the detection rate and CIC proportion are not very high, the specificity is high, irrespective of tumor type. Cell cannibalism can also function as a cytological indicator to diagnose squamous cell carcinoma (SCC) of the breast (44). In conclusion, CIC can be used as a reliable cytomorphological indicator for the diagnosis of malignant tumors (**Figure 5**).

### 4.2 Prognostic significance

Prognostic markers are crucial to accurately developing individualized therapies for cancer patients, thereby reducing long-term costs and patient distress. The prognosis of tumors plays an important role in guiding clinical treatment, and the application of prognostic factors of tumors for tumor treatment can improve patient prognosis. The occurrence and development of tumors involve a series of complex processes. Because several factors affect the prognosis of tumors, the development of novel biomarkers is required to accurately predict the prognosis. In recent years, CIC has emerged as a complex behavior of cancer cells; therefore, an increasing number of investigations is being conducted to study its effect on clinical prognosis. The results of clinical investigations into the role of CIC in cancer patient prognosis are summarized in **Table 1**. Heterotypic CIC was identified as an unfavorable prognostic factor for pancreatic cancer (58), breast cancer (46), non-small cell lung cancer (56), and buccal mucosa SCC (50). Interestingly, a highly heterotypic CIC phenomenon was found to be positively correlated with longer survival in patients with hepatocellular cancer (54). This association can be attributed to the effective tumor cell-killing ability of NK cells residing within the heterotypic CIC structure. Homotypic CIC was found to be significantly associated with the poor prognosis of breast cancer (47, 48), lung adenocarcinoma (55), pancreatic cancer (59), hepatocellular cancer (53), rectal cancer (45), bladder cancer (43), head and neck SCCs (45, 52), and early-stage oral tongue cancer (57). However, in patients with esophageal SCC (51), anal cancer (45), and early breast cancer (46, 49), homotypic CIC showed a better prognosis. This discrepancy could be resolved by focusing on the mechanisms underlying CIC formation or the differences in host and target cells, which could create a heterologous population of CICs. Homotypic CIC is also positively correlated with KI-67, a prognostic factor in breast cancer, which shows a high level at higher tumor, node, and metastasis (TNM) stages (47). Therefore, it can be speculated that homotypic CIC could indicate a more unfavorable prognosis for patients with breast cancer at more advanced stages. However, in patients with early-stage breast cancer, homotypic CIC is prognostically favorable for patient survival (46, 49); this finding is consistent with the tumor-suppressing role of entosis. On the basis of these studies, we suggest that homotypic CIC may inhibit tumorigenesis in the early stages of tumor development; however, in the late stages of tumor development, it may promote tumor progression. In summary, the role of CIC as an independent prognostic factor in cancer indicates that CIC could serve as a novel functional marker and, along with classical pathological factors, improve prognostic prediction for cancer patients.

**Table 1:** Implication of cell-in-cell (CIC) for clinical prognosis in different studies.

Tumor type		CIC category	Prognostic significance	Reference
Anal cancer		Homotypic	**Entosis is associated with a better prognosis in anal carcinomas**	(45)
Bladder cancer		Homotypic	Homotypic cannibalism was an independent factor in the prediction of the progression	(42)
Breast cancer		Heterotypic	MiT was identified as a potent adverse prognostic marker	(46)
		Homotypic	**Higher TiT predicted longer patient survival**	(46)
		Homotypic	Entosis did not depend on the hormonal status of the cancer but correlated with two prognostic factors: Ki67 and HER2	(47)
		Homotypic	A high frequency of CIC predicted poor survival	(48)
		Homotypic	**CICs were prognostically favorable for local recurrence-free survival and disease-free survival**	(49)
Buccal mucosa squamous cell carcinoma		Heterotypic	The frequency of NiT as a novel predictor was significantly negatively associated with both recurrence-free survival and disease-specific survival	(50)
Esophageal squamous cell carcinoma		Homotypic	**TiT tended to demonstrate better prognostic performance in patients at later TNM stages or T stage**	(51)
Head and neck squamous cell carcinoma		Homotypic	Adverse prognosis of patients with homotypic CIC occurrences	(52)
		Homotypic	Large numbers of CIC events showed adverse effects on patients’ survival	(45)
Hepatocellular cancer		Homotypic	Homotypic CIC predicted poor survival of patients	(53)
		Heterotypic	**High CIC formation was positively correlated with longer survival**	(54)
Non-small cell lung cancer		Homotypic	Entosis was an independent predictor of poor outcome and disease recurrence	(55)
		Heterotypic	Heterotypic CICs were associated with adverse prognoses for patients	(56)
Oral tongue cancer		Homotypic	Homotypic CICs were significantly associated with a higher rate of cancer-related mortality	(57)
Pancreatic cancer		Heterotypic	LiT and MiT were identified as potent adverse prognostic markers impacting young female patients with early-stage pancreatic cancer	(58)
		Homotypic	Entotic CIC was independently associated with a poor prognosis	(59)
Rectal cancer		Homotypic	Large numbers of CIC events showed adverse effects on patients’ survival	(45)

CIC, cell-in-cell.; CICs, cell-in-cell structures; LiT, lymphocyte in tumor cell; MiT, macrophage into tumor cell; NiT, neutrophil-in-tumor structure; TiT, tumor cell into cell; TNM, tumor, node, metastasis. Bold font indicates the favorable effect of CIC on prognosis.

## 5 CIC as an emerging concept in anticancer therapy

Immunotherapy, chemoradiotherapy, and targeted therapy have been widely used in tumor management strategies, including both systemic and local treatment methods. Cancer recurrence and the resistance of cancer cells to anticancer drugs are important causes of treatment failure and remain major challenges for anticancer treatment (9). Overcoming these challenges is essential to achieve long-lasting and effective cancer treatment and to prevent cancer recurrence. The mechanisms of anticancer-drug resistance and cancer recurrence have been investigated but have not yet been fully characterized.

The internalized cells in homotypic CIC can escape in an intact state after entering the cells; this feature may afford protection to the internalized cells in an unfavorable therapeutic microenvironment, such as exposure to a chemotoxic drug and immune surveillance. Because immune checkpoint inhibitors have shown limited effects in most mouse models, Kellie Smith et al. (60) conducted *in*-*vivo* experiments where human breast, colon, and melanoma tumor cells were incubated with pre-activated allogeneic T cells. The results demonstrated that the majority of individual cells underwent cell death whereas surviving tumor cells formed organized CIC structures. *In*-*vitro* studies on melanoma tumors treated with autologous reactive T-infiltrating lymphocytes revealed a high frequency of CIC formation in relapse tumors. This is due to the induction of phosphorylation in tumor cells by cytotoxic T-cell immunotherapy, which specifically targets the transcription factors signal transducer and activator of transcription 3 (STAT3) and early growth response-1 (EGR-1) and subsequently promotes the formation of CIC. Therefore, to mitigate tumor resistance toward subsequent drugs, it is recommended that these factors are inhibited prior to immunotherapy. Similarly, in the targeted treatment of prostate cancer with nintedanib (61), prostate cancer cells acquired resistance to nintedanib and survived by activating entosis formed by the upregulation of E-cadherin and ROCK1/2 through the PI3K/CDC42 signaling pathway. Thus, during treatment with anticancer drugs, cancer cells hide inside each other to avoid destruction. Radiotherapy and chemotherapy can destroy tumors by inducing apoptosis and necrosis in tumor cells. Radiotherapy and chemotherapy can also induce tumor cells to enter a senescence state, that is, move from cell cycle arrest into cellular quiescence (62). Doxorubicin induces senescence in p53 wild-type breast cancer cells, leading to the acquisition of a senescent phenotype. These senescent cells exhibit a significant enrichment for genes related to macrophage and phagocytosis, and efficiently engulf neighboring senescent or non-senescent tumor cells at a high frequency, forming numerous CICs. The target cells that undergo processing in the lysosome and subsequent breakdown confer an advantage to senescent host cells, which promotes the viability of senescent cancer cells. This ultimately leads to tumor persistence and poor patient survival (63). With this perspective in mind, Gottwald et al. (64) discovered a significant increase in CICs in rectal cancer tissues after neoadjuvant radiochemotherapy (RCT) compared with pre-RCT conditions. In addition, a notably high presence of senescent cells and CICs is associated with unfavorable clinical outcomes following RCT. It is speculated that CICs formed by senescent cells enhance cell viability and re-enter the cell cycle during antitumor treatment, thus providing additional energy and material for tumor recurrence and growth. This may be the cause of tumor recurrence and drug resistance during radiotherapy and chemotherapy.

Heterotypic CIC is formed by tumor cells and immune cells. In the tumor microenvironment, although NK cells and CD8^+^ T cells can kill tumor cells, they can also become engulfed by tumor cells, leading to their degradation and immune escape. Yun-Jeong et al. (25) have demonstrated a positive correlation between anticancer drug resistance and heterotypic cancer-initiating cell structures in EGFR-overexpressing lung cancer cells. The heterotypic CICs formed by non-small-cell lung cancer (NSCLC) cells and NK cells exhibited a higher proliferative capacity and more malignant phenotype than the non-CIC NSCLC cells. Following immunotherapy with NK-cell therapy and chemotherapy with cisplatin, doxorubicin, gemcitabine, etoposide, and docetaxel treatment, heterotypic CICs displayed lower sensitivity to NK cytotoxicity and greater resistance to anticancer drugs than non-CIC cancer cells.

Taken as a whole, the CIC phenomenon can be considered a mechanism of drug resistance and used to predict whether a patient would respond to anticancer therapies or show resistance to anticancer drugs.

## 6 Conclusion and perspectives

Various studies have reported and highlighted the phenomenon and effect of CIC in tumors. Here, we discussed the mechanism of occurrence of two subtypes of CIC and the role of CIC in tumors. CIC has been frequently observed in various human tumor tissues (40). Thus, it can be considered a common feature in the tumor development process. We propose that CIC could be a new tumor marker that affects the prognosis and anticancer therapy of tumors. CIC formation can provide nutritional support to tumors, induce genomic instability to further promote a malignant phenotype, select populations with more survival advantages, suppress the immune microenvironment, evade immune cell attack, and promote tumor development and metastasis. Entosis can also act as a mechanism to eliminate abnormal cells, such as those with abnormal mitosis and loss of adhesion; thus, it could play a role in tumor suppression. We predict that entosis, similar to autophagy, has a dichotomous role; it plays contrasting roles at different times and in different contexts of tumors (65). Entosis can perform an inhibitory function by eliminating abnormal cells in tumorigenesis and the early stage of tumor development. As the tumor progresses, tumor cells use entosis to adapt to different environments, such as nutrient-replete conditions or the tumor immune microenvironment, thus promoting further tumor development.

Although we have a preliminary understanding of the mechanism of CIC formation, several questions that have not yet been answered remain. Deciphering these questions will enable us to further understand the role of CIC in clinical treatment. First, before the initiation of homotypic CIC formation, the mechanism for determining the identity of host and target cells remains unclear. It is currently believed that the degree of stiffness determines the tumor cells that will engulf or become engulfed (66). KRAS (39), c-Myc (67), mutant P53 overexpression (68), loss of CDKN2 (69), and CD44 downregulation (54) confer a “host” status to the tumor cells by making them softer. However, current knowledge of mechanisms of engulfment appears to be insufficient. Studies on CIC formation indicate that the stiffness of cells might not be the only factor leading to the invasion of soft cells and that other sophisticated mechanisms manipulating this process are present in tumor cells. For heterotypic CIC, the mechanisms responsible for the inversion of the host–target relationship between tumor cells and immune cells remain unknown. Second, based on the formation process, CIC can be classified into two types: (1) target cell-initiated and (2) host cell-initiated. The former entails the active invasion of host cells by target cells, and the latter entails the active engulfment of target cells by host cells. We predict that the formation of CIC requires the combined efforts of both the host and target cells, that is, the active engulfment of host cells and the cell entry momentum provided by target cells. Moreover, under normal conditions, only specialized phagocytes have the ability to phagocytose. The mechanism of acquisition of phagocytic capability by tumor cells remains undefined. Cell fusion can lead to gene transfer, and tumor cells may acquire the ability to phagocytose through fusion with macrophages. The most critical limitation is the lack of specific markers for differentiating homotypic cell cannibalism and entosis from heterotypic cell cannibalism and emperitosis in tumor tissues; this limitation should be promptly addressed for a greater understanding of the CIC phenomenon. Finally, due to the lack of a unified and specific counting method, CIC counting currently relies on manual counting, which may cause bias in the results. Tang et al. (70) proposed an automated identification method for specific CICs. Uniform high-throughput counting methods such as this are needed to improve the reliability of research results and provide more precise data support for clinical treatment.

Based on current studies, CIC can be considered a potential biomarker for tumor prognosis and resistance to anticancer agents. The anticancer treatment process can induce CIC formation and cause treatment recurrence and resistance. Thus, targeting CIC could open new avenues for cancer therapy. In the future, more large-scale studies on a variety of cancers are required to provide a more reliable basis for CIC formation and its role in tumor development.

## Author contributions

Conceptualization: XL and JY; writing and original draft preparation: XL; writing, review, and editing: JY. All authors contributed to the article and approved the submitted version.
